# Scenario driven data modelling: a method for integrating diverse sources of data and data streams

**DOI:** 10.1186/1471-2105-12-S10-S17

**Published:** 2011-10-18

**Authors:** Shelton D Griffith, Daniel J Quest, Thomas S Brettin, Robert W Cottingham

**Affiliations:** 1 Biosciences Division, Oak Ridge National Laboratory, Building 1059, P.O. Box 2008, MS 6420, Oak Ridge, TN 37831-6420, USA

## Abstract

**Background:**

Biology is rapidly becoming a data intensive, data-driven science. It is essential that data is represented and connected in ways that best represent its full conceptual content and allows both automated integration and data driven decision-making. Recent advancements in distributed multi-relational directed graphs, implemented in the form of the Semantic Web make it possible to deal with complicated heterogeneous data in new and interesting ways.

**Results:**

This paper presents a new approach, scenario driven data modelling (SDDM), that integrates multi-relational directed graphs with data streams. SDDM can be applied to virtually any data integration challenge with widely divergent types of data and data streams. In this work, we explored integrating genetics data with reports from traditional media. SDDM was applied to the New Delhi metallo-beta-lactamase gene (NDM-1), an emerging global health threat. The SDDM process constructed a scenario, created a RDF multi-relational directed graph that linked diverse types of data to the Semantic Web, implemented RDF conversion tools (RDFizers) to bring content into the Sematic Web, identified data streams and analytical routines to analyse those streams, and identified user requirements and graph traversals to meet end-user requirements.

**Conclusions:**

We provided an example where SDDM was applied to a complex data integration challenge. The process created a model of the emerging NDM-1 health threat, identified and filled gaps in that model, and constructed reliable software that monitored data streams based on the scenario derived multi-relational directed graph. The SDDM process significantly reduced the software requirements phase by letting the scenario and resulting multi-relational directed graph define what is possible and then set the scope of the user requirements. Approaches like SDDM will be critical to the future of data intensive, data-driven science because they automate the process of converting massive data streams into usable knowledge.

## Background

Next generation DNA sequencers and online social media produce a data deluge requiring new tools for storage, representation, visualization, querying, interaction, and integration [1]. Data streams are leading to interesting observations. Google used a single information source, the search logs of terms entered by users, to predict a recent flu outbreak nearly two weeks ahead of the CDC [2]. Developing more powerful methods requires integration of multiple information sources and connecting it in ways that are timely, relevant, and capable of distinguishing useful information from noise. Some of these capabilities have been explored in simulation systems that model the spread of disease [3]. There is a pressing need for a bio-security system that will do this in near real time.

### BioSITES

The Biological Signature Identification and Threat Evaluation System (BioSITES) is a prototype effort to develop a secure, authoritative, predictive and complete reference standard for bio-threat detection and mitigation that will support detection R&D and lead to near real-time bio-surveillance and bio-threat identification. BioSITES is currently in incubation status at Oak Ridge National Laboratory; the work discussed in this paper is part of the BioSITES effort.

BioSITES implements methodologies that deal with complex bio-surveillance data integration requirements. No single institution owns all of the data necessary for bio-surveillance. It is highly heterogeneous and requires petabytes of storage space. The BioSITES knowledgebase is being constructed assuming that the data infrastructure will be composed of multiple distributed and interoperable data repositories serving as reference catalogs. This work is mainly focused on the principles for constructing a semantic knowledgebase capable of integrating diverse data repositories and data streams. The section titled ‘The BioSITES Streaming Data Kernel’ provides an overview of how data streams through the BioSITES system and clarifies requirements of the BioSITES knowledgebase. A more thorough discussion of the streaming kernel is the topic of a pending publication.

### The data integration challenge

Bio-surveillance requires rapid integration of vastly different types of information. Resources such as the Antibiotic Resistance Genes Database [4], MvirDB [5], SuperToxic [6], and PIG – the pathogen interaction gateway [7] are all excellent examples of molecular catalogues used in bio-surveillance applications. These repositories can be combined with geo-location, time and important information from the literature to form a model of the entities and relationships involved as a disease spreads. A key challenge is to codify this heterogeneous data and information in a computationally useful manner that meets bio-surveillance requirements. The first step to doing this is to integrate the data in a semantically consistent data model.

In bioinformatics, dominant approaches to heterogeneous data integration include: (1) an operational data store such as Chado [8] – where heterogeneous data is integrated into a unified model by importing each data source into a unified relational schema, and (2) ‘knuckles-and-nodes’ [9], where data is distributed and integrated through the use of ontologies. BioSITES requires the ability to integrate heterogeneous data streams, and distributed reference catalogs, making an ontology centric approach more appropriate.

The knuckles-and-nodes approach is also used in the Semantic Web [10]. The Semantic Web is to data what the World Wide Web is to documents; a globally linked data store where data elements are distributed across multiple servers on the Internet. The Semantic Web’s representation is a multi-relational directed graph (MRDG) implemented through the resource description framework (RDF) [11,12]. A large part of the Semantic Web, relevant to genetics and biology has been constructed by the Bio2RDF project [13] and this vast resource was used in the construction of BioSITES.

This article introduces scenario driven data modelling (SDDM). SDDM is a data integration process that defines the required relationships between diverse sources of data, data streams, and software components. SDDM is closely related to the concept of data mashups or “purpose driven, customized data integrations that facilitate question answering on a topic of interest” [14]. Scenarios (defined further in the section ‘Scenarios’) are like mashups, they are purpose driven and use similar integration strategies, but are larger in scope and complexity because they include analytical methods and software components designed for analysing data that exists outside the Semantic Web. These analytical methods and software components analyse data as a stream, and when they find matches, they integrate data into the Semantic Web.

### Significance

People consume content on the web. To navigate the web, a person starts with one article, and then navigates to other articles; building an interpretation of the world as they follow hyperlinks, consume additional content, and perhaps ‘teleport’ randomly to other articles and topics. Data is presented on the web when it is imbedded into human readable HTML or images and served on request. As the amount of data available is exploding, it is increasingly likely that people traversing the web will miss important content. Machines need to be used to help augment human search capabilities and reduce the time it takes to find and communicate important information. However, with data represented in free text, machine interpretation is limited because we have not discovered a general-purpose way to compile natural language down to a level understood by computer programs. It is however possible to write simpler software with very limited goals that has a limited understanding of free text - search engines being good examples.

Before delving into the details of the SDDM process, we will first discuss the significance of using the Semantic Web as a platform for bio-defense. Internationally, professionals and researchers are currently developing assays, assembling information, coordinating prevention efforts, notifying first responders and the media, and identifying and characterizing new pathogens. While each of these efforts has merit, specialization tends to create silos where individuals who need to communicate with each other may not connect because they have not each identified the value in the connection. A top down solution to this problem is to build cross-cutting communities focused on integrative approaches. A bottom up approach to the problem uses social networks to connect individuals and focus on specific problems. Both of these approaches are important, but focus on solving integration problems through people and personal connections. In both approaches, software systems are isolated systems, integration of information takes place in the humans who use these systems. Near real time bio-threat prevention and detection requires integrated software systems.

A more powerful alternative approach to isolated systems is an integrated and global platform that includes the Semantic Web and its capabilities to ascribe meaning to and draw inferences from data. SDDM allows automated integration methods that becomes extremely useful when dealing with large, complex streaming data that otherwise would not be humanly possible to accurately and reliably integrate. SDDM enables distillation of massive data amounts and computing of analyses into a set of facts and a resolution of their accuracy for decision making. The RDF data representation also accommodates changes in representation and ownership beyond what has been previously possible.

## Results

Utilizing SDDM, we defined and constructed a system that integrates heterogeneous data based on a bio-surveillance scenario that operates in near real-time, meeting scenario requirements. The SDDM process and the example of its implementation presented here, represents an iterative refinement of a data integration process. SDDM combines data feeds, private and public data stored in files or databases and the Semantic Web. SDDM starts with a scenario, or free text description, and then gradually updates the scenario into a multi-relational-directed graph encoded in RDF. Data stored in flat files or databases is integrated by constructing software called RDFizers (see methods and glossary) that convert traditional data representations into multi-relational-directed graphs (a collection of RDF statements). RDFizers were built for ProMed to link molecular concepts to time and geo-location. Resources available on the Semantic Web (e.g. Bio2RDF) were used to link molecular concepts to DNA and protein sequences.

The SDDM process also integrates streaming data. The resulting software prototype enables streaming data from genetic sequencers to be compared to sequences identified in Bio2RDF, and, when a match is found, RDF statements integrate the new findings into the Semantic Web. This data integration approach was applied to the construction of a near real-time and completely automated analysis system capable of monitoring the NDM-1 gene globally. This proof of concept further illustrates the power and flexibility of ontology-centric data integration approaches.

### The BioSITES streaming data kernel

**Figure 1:**
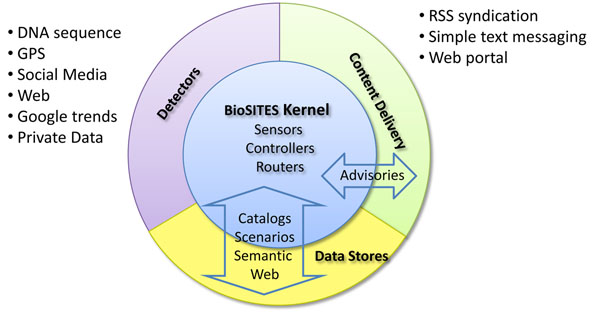
**A modular view of the BioSITES architecture.** Subsystems (Detectors, Data Stores, Content Delivery, and the Kernel) are all required to realize systems for monitoring streaming data. The BioSITES Kernel is at the heart of the architecture, providing monitoring capabilities. Each other subsystem requires a functioning kernel to run. Subsystems interact with the BioSITES kernel through clearly defined interfaces.

The BioSITES streaming kernel looks at feeds of data and attempts to match elements in the knowledgebase to data in the stream. If data in the stream is matched to specific elements in the catalog, then an advisory is published to alert first responders and analysts. These advisories feed the decision support system. The system architecture consists of four subsystems: data feed generation (detectors), data stores (catalogs), content delivery (advisories) and the streaming kernel. Figure 1 illustrates a module view of the BioSITES architecture that shows the system’s principal components. BioSITES detectors feed a massive amount of data continuously to the BioSITES system. Example feeds include web crawlers, DNA sequencing machines, logistics information, weather sensors, and satellite imagery. As this information streams past the BioSITES system, useful connections and knowledge are automatically extracted and then integrated directly into the Semantic Web or presented to users.

Although there are many combinations of data important in situational awareness and response, this article focuses on data integration dealing with time (date the events took place), space (geo-location of where the event occurred) and genetics (the molecular components of the outbreak/attack).

### Scenarios

**Figure 2:**
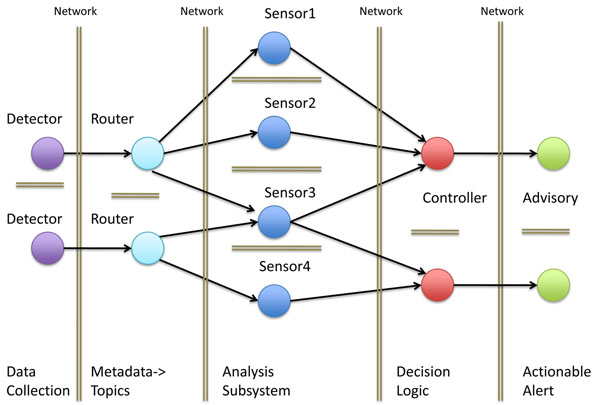
**A runtime view of the BioSITES system. BioSITES is achieved by connecting software components (the circles in the figure) from multiple institutions.** These software components are connected via the message oriented middleware (MOM), ActiveMQ. MOM uses the publish-subscribe pattern to connect software components. Data is forwarded from one component to another when the second component subscribes to the first. BioSITES components include detectors (e.g. DNA sequencers and web crawlers), Routers (components that route data appropriately in the system based on the metadata), Sensors (analysis routines or workflows that identify and filter important signals in the data stream), Controllers (extensions of the Semantic Web that contain decision logic to process and integrate workflow outputs), and Advisories (components that contain reporting capabilities for displaying and relaying information). The double lines in the figure illustrate network boundaries. Data storage and access in the system is constrained by these network boundaries. Each component of the system is aware of all upstream components through messages that flow through the system.

A BioSITES scenario is a structured description of a malicious action or series of actions that causes harm or disruption in health, the economy, or day–to-day activities in people, crops or livestock. A scenario could be based on real world events or be hypothetical. A scenario need not be confined to a specific organism or spread mechanism, but these could be attributes of a specific scenario. Outside of the bio-security arena, a scenario could be used to improve public health by addressing naturally occurring pathogens. A scenario identifies the downstream data users and uses. These drive data collection and refinement requirements. BioSITES scenarios are concerned with identifying data streams that provide temporal, spatial and sequence information when combined. A scenario is used to scope the software development process. For example, a scenario requires one or more catalogs that are later used by sensors of the BioSITES kernel (Figure 2). A scenario also identifies possible data streams that will be monitored and algorithms that can be used in monitoring those data streams. A scenario may be extremely specific or more general in the way that it references data stream elements and catalogs. For example, a scenario could state ‘a toxin was used’ instead of ‘Botulinum toxin was used’.

A scenario begins as a free text document. An expert constructs a scenario, integrating the sensitivity and specificity of each data source. This results in an integration of information that is not misleading to users. Elements in scenario documents map directly to software components used in the BioSITES streaming kernel. Each scenario document corresponds one-to-one with a BioSITES controller responsible for generating advisories related to that scenario. The controller is a state machine that when it finds all of the conditions required (published by sensor algorithms) in the scenario, it publishes an advisory and integrates information into the BioSITES data store.

Molecular biology is just too complex to cover every possibility. Simple scenarios give us a starting point and basic capabilities. These capabilities can be leveraged later to discover trends and patterns present in multiple scenarios.

### The BioSITES data store

**Figure 3:**
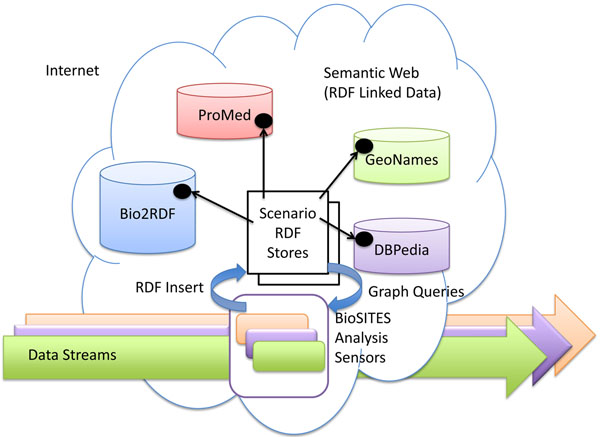
**A conceptual view of where data is stored in the BioSITES data store.** Data in streams is analysed in near real time and is not stored. RDF Statements found on the Semantic Web are each stored in repositories owned by each institution. Portions of these repositories may also be cached in the Scenario RDF stores to improve performance. RDF Statements created by analysis are stored in RDF Stores distributed across the BioSITES system, with each store corresponding to one or a few scenarios. Catalogs required by a scenario (e.g. a set of sequences) must be converted to RDF, and then made available for traversal by software.

The BioSITES Data Store (Figure 3) combines data that exists inside the Semantic Web and data available on the Internet. Data available on the Internet is handled as data streams that flow through BioSITES analysis routines (or BioSITES Sensors). These analysis routines compare a reference catalog to data found in the stream. If a match is found, it forwards the information to the controller. The controller can then choose to integrate the structured information delivered by the sensor into a local RDF model that represents our current understanding of events, genetic elements, places, people and other variables. This RDF model is connected, via links to other resources on the Semantic Web, so as other information is deposited, the RDF model improves.

### Example of SDDM applied to a global health threat

This section provides a demonstration of the SDDM approach, building a multi-relational directed graph about the NDM-1 gene [15-17] that confers antibiotic resistance to some of the most powerful antibiotics and is an emerging global health threat. A scenario was constructed based on the publication by Kumarasamy et al. [15], “Emergence of a new antibiotic resistance mechanism in India, Pakistan, and the UK: a molecular, biological, and epidemiological study”.

#### SDDM step 1: a scenario is selected for refinement

In the first step Kumarasamy et al. is analyzed and specifics are added about the genetics of NDM-1 (based on searches of NCBI and ProMed [18]), times when NDM-1 has appeared, and geo-locations where NDM-1 has appeared, to construct the following scenario:

ANTIBIOTIC RESISTANCE GENE NDM-1 LOCATED ON GRAM NEGATIVE PLASMID

*Gram-negative Enterobacteriaceae with resistance to carbapenem conferred by New Delhi metallo-β-lactamase 1* (*NDM-1*) *is a public health threat.*

*“Resistance to beta-lactam antibiotics has become a particular problem in recent decades*, *as strains of bacteria that produce extended-spectrum beta-lactamases have become more common. These beta-lactamase enzymes make many*, *if not all*, *of the penicillin and cephalosporin ineffective as therapy. Extended-spectrum beta-lactamase–producing E. coli are highly resistant to an array of antibiotics and infections by these strains are difficult to treat. In many instances*, *only two oral antibiotics and a very limited group of intravenous antibiotics remain effective. In 2009*, *a gene called New Delhi metallo-beta-lactamase* (*shortened NDM-1*) *that even gives resistance to intravenous antibiotic carbapenem*, *were discovered in India and Pakistan in E. coli bacteria. NDM-1 is known to exist on a plasmid and has been transferred across bacteria via horizontal gene transfer.*

*Increased concern about the prevalence of this form of "superbug" in the United Kingdom has led to calls for further monitoring and a UK-wide strategy to deal with infections and the deaths. Susceptibility testing should guide treatment in all infections in which the organism can be isolated for culture.”*[15]

• BioSITES Detector 1 (data source 1): First responders/clinicians isolate the laboratory strain using the techniques outlined in the CDC manual [19]. When the patients remain ill, the samples are sequenced with a 454 pyrosequencer. geo-location of where the sample was obtained is imbedded in metadata accompanying the sample. Note, this includes all 454 runs taken from the sick, not just E. coli infections.

• BioSITES Detector 2 (data source 2): As new ProMed articles are published, software serializes the articles and streams them through the BioSITES Kernel. Note these articles may not relate to NDM-1.

• BioSITES Data Catalog_1 (reference data): Three representative sequences were found at NCBI with GI numbers: 300422615, 255031061 and 302826879.

• BioSITES Data Catalog_2 (reference data): Occurrence of NDM-1 can be found in historical ProMed articles: 20100815.152812, 20100817.2853, 20100914.3325, 20101005.3604, and 20101028.3908.

• BioSITES Sensor Method_1 (analytical technique 1): A nucleotide level BLAST (at significance level 1e-15) for detecting a match between CATALOG_1 and the sequence reads from DETECTOR 1. The blast alignment must match the region of the sequence corresponding to the NDM-1 gene.

• BioSITES Sensor Method_2 (analytical technique 2): GPS coordinates are obtained for the source location of the sample and imbedded in the metadata of read sets from sequencing machines. Each sample obtained will be scanned to identify a match to positions in CATALOG1003_2. If the GPS coordinates do not match coordinates in the existing catalog, then this indicates a possible new location where NDM-1 has spread.

• BioSITES Sensor Method_3 (analytical technique 3): ProMED mail articles are scanned using Data Catalog_2 for additional content relating to NDM-1.

#### SDDM step 2: create first iteration of RDF multi-relational directed graph

**Figure 4:**
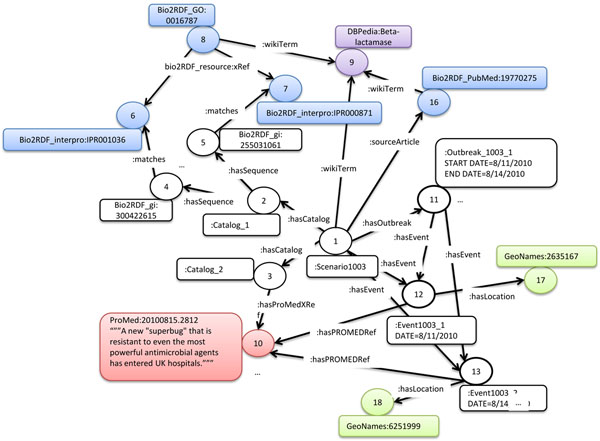
**A simplified view of the results from the SDDM process.** Five different ontologies are used in the construction of this multi-relationship graph: the Scenario Ontology (abbreviated with a colon, :), Bio2RDF (abbreviated with Bio2RDF_X where X is a namespace tag e.g. if X = PubMed, then Bio2RDF_PubMed = http://bio2rdf.org/pubmed), GeoNames, ProMed, and DBPedia. The SDDM process created all edges in the example, and all nodes in black and white. Bio2RDF (Blue), GeoNames (Green), and DBPedia (Purple) nodes already existed on the Semantic Web. Red nodes representing ProMed mail articles are integrated using an RDFizer as part of the SDDM process. The NDM-1 example created RDF individuals corresponding to Wikipedia articles, InterProScan models, PubMed articles, ProMed articles, Gene Ontology terms, a scenario, catalogs, events, outbreaks, NCBI sequences (gi numbers), and GeoName locations. The nodes and edges diagrammed in this figure are physically stored in 5 locations: Bio2RDF nodes marked in blue are stored on the main Bio2RDF server or an acceptable mirror, GeoNames nodes marked in green are stored on the GeoNames infrastructure, DBPedia nodes marked in purple are stored on the DBPedia servers or a mirror, ProMed nodes marked in red are stored in a local graph database (Neo4j), and nodes and relations identified in the scenario, marked in black, are stored in a local RDF database on the machine where the scenario is built.

Step 2 of the SDDM process focuses on conversion of the scenario into a multi-relational directed graph. To convert the scenario into a MRDG, BioSITES scenario ontology and Protégé [20] are used to re-write the scenario into a RDF/XML document. Creation of the RDF graph begins with a single node, representing the BioSITES scenario document (Figure 4, node 1). RDF statements then connect the BioSITES scenario to resources on the Semantic Web. This example scenario is based on the PubMed article 19770275, so the Scenario:sourceArticle property was used to connect the scenario object to the Bio2RDF_pubmed:19770275 article (Figure 4, node 16). This indirectly links MESH terms [23], GO terms [24], PubMed articles [22] and similar entities already connected via the Bio2RDF project. These terms and articles can be used to find scenarios that are related. This multi-relational directed graph is not a static entity, as more information is identified; the graph is updated (See step 3, 4 and 5). This multi-relational directed graph links resources already on the Semantic Web into a data structure that is used by BioSITES sensors. The result at this stage is a minimal graph in that it only represents what was included in the scenario with direct links to extermal resources on the Semantic Web. The multi-relational directed graph constructed in this step of the SDDM process links location (GeoNames [21]), Dates (scenario:events), and molecular objects (Bio2RDF [13] resources).

#### SDDM step 3: identify and convert to RDF those resources required by the scenario but absent in the Semantic Web

The construction of the two catalogs described in the scenario, NDM-1 genes and ProMed articles related to NDM-1 (Catalog_1 and Catalog_2 respectively), illustrates two common situations. The first situation links elements in the scenario document to elements that already exist (or will soon exist via a 3^rd^ party update) in the Semantic Web using RDF statements. The second situation involves creating new Semantic Web content by RDFizing data represented in traditional structures and then linking the scenario document elements to the newly created Semantic Web content.

In this example, a search was used to identify examples of the NDM-1gene. The current Bio2RDF release (August 2010) did not contain annotated sequences for the GI numbers outlined in the scenario. This required the construction of Bio2RDF entries for each of the sequences identified in the scenario. The Bio2RDF software suite was used for the conversion [13]. An RDF catalog (Figure 4 node 2) was created to link the scenario object to each known instance of the NDM-1 gene (Figure 4 nodes 4,5,…). InterProScan was used to connect each Bio2RDF_gi instance linked by Catalog_1 to functional annotation on the Semantic Web (Figure 4 nodes 6,7,8,…). Functional annotation properties include gene ontology terms, HMMer [25] profiles, and scientific terms. This functional annotation connects entities identified in this scenario to catalogs used in other scenarios that have similar functional annotation. In the future, this will help generalize scenarios and perhaps even automatically generate them. For example, NDM-1 is annotated as a Beta-lactamase, which based on sequence similarity, matches several entries in the MvirDB database [5]. MvirDB is applicable in many scenarios involving category A agents.

A second catalog, data source 2, (Figure 4, node 3), was constructed to represent historical articles from ProMed. An RDFizer was constructed to convert historical ProMed articles into RDF. For each article, we extracted the article ID, date, subject line and text, and populated a simple RDF graph. ProMed articles referencing NDM-1 were mined to identify the time and location of NDM-1 events. The technology does not yet exist to reliably extract locations from the articles and associate them with events, so manual extraction of the locations for those articles relating to NDM-1 was required. Three rdfs:classes where used in construction of the multi-relational directed graph representation for the disease spread described in ProMed; Scenario:event, Scenario:outbreak, and GeoNames:Feature. These rdfs:classes [26] correspond to time (Scenario:event), time intervals (Scenario:outbreak), and geo-location (GeoNames:Feature/GeoNames:Location). A Scenario:event describes a real world event as it is described in ProMed. Data associated with this event (e.g. the time and date) are embedded as rdfs:Literals [26] and associated with the event object. Concepts described in the event such as symptoms, locations, genetic sequences and other articles are described by Resource Description Framework Schema (RDFS) [26] statements that link the Scenario:event to rdfs:Resources on the Semantic Web. Figure 4 illustrates how events are linked to locations, using the rdfs statement <Scenario:Event1003_1 Scenario:hasLocation GeoNames:2635167>. GeoNames:2635167 represents the United Kingdom. GeoNames maintains synonyms for this location, GPS coordinates, elevation, population, and other geographic specific information. Scenario:outbreaks are used to connect sets of Scenario:events. A Scenario:outbreak contains events that co-occur in time and have an occurrence of a disease/infection greater than would be expected in an area. Scenario:outbreak objects are used in the system to model disease spread over time and associate disease spread with genetic variation. Figure 4 shows a simplified version of the multi-relational directed graph constructed as a result of steps 2 and 3 of SDDM process.

#### SDDM step 4: identify data feeds

This set of the SDDM process primarily focuses on the identification of data sources (often near-real time data streams). In the NDM-1 scenario, we identified the Sequence Read Archive at NCBI (http://www.ncbi.nlm.nih.gov/sra) as a source of genetic information (serving as a proxy for high throughput sequence data), and ProMed [18] mail as a source for geo-location and time based information. For the sequence data, we implemented a BioSITES detector that monitors NCBI Entrez for new or updated submissions to the Sequence Read Archive (SRA). If a new or updated submission is detected, reads from the submission are streamed across the network to every computer running a sensor that subscribes to the SRA BioSITES detector. A program to automatically download new ProMed articles as they are published and stream them through the BioSITES kernel was developed. Articles in the ProMed data stream are not automatically converted to RDF in the streaming process. However, if a new article matches a catalog in the BioSITES system (i.e. if the ProMed article has content relating to NDM-1) , then relevant elements of the article are extracted and represented in RDF. This process, although subjected to some automation, was mostly a manual effort.

#### SDDM step 5: identify analytical routines for comparing information in the data stream to the concepts outlined in the scenario

**Figure 5:**
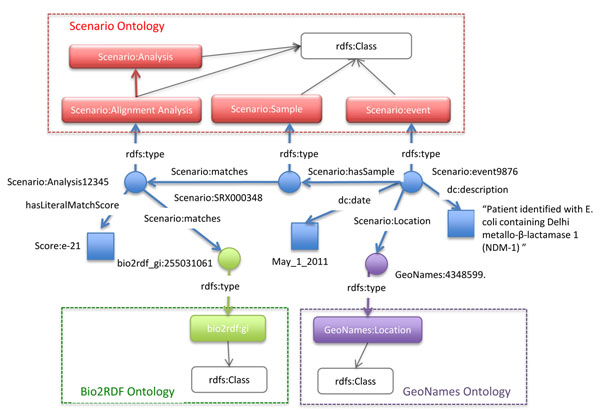
**A multi-relational multi-ontology directed graph representing the output from an analytical routine (BioSITES sensor).** RDF Statements represent the results from an analysis (statements are each edge in blue). Statements are also made between entities of the multi-relational directed graph, Literals (blue squares) and ontologies (Scenario, bio2rdf, and GeoNames). Ontologies (dashed rectangles) also contain relationships between classes (rounded rectangles). An analysis is responsible for linking bio2rdf entities to samples. Samples correspond to events and events have locations.

Scenarios require the use of one or more sensors to analyze streams of data coming from detectors. In the NDM-1 scenario, the sensor (Sensor_1) was defined to compare sequences in a data stream to the NDM-1 catalog (Catalog_1) based on alignment. In this case, we used the BioSITES BLAST sensor to match reads in the read set to sequences in Catalog_1 (e value -15). When a read set matches a sequence in Catalog_1, RDF statements describing the match can be added to the multi-relational directed graph (Figure 5).

The NDM-1 scenario also required the construction of an additional sensor (Sensor_2) to compare global positions and text in ProMed articles (Catalog_2). Newly published ProMed articles are scanned to identify Delhi metallo-β-lactamase 1 (NDM-1) or close synonyms. If the article matches, it is then scanned for location matches in the GeoNames database. All matches are geo-coded and associated with one or more new events. This information is serialized to the NDM-1 controller where it is converted into RDF.

#### SDDM step 6: based on the scenario and the resulting MRDG, identify the data outputs that meet end-user decision support needs and define queries to produce those outputs

The last step in an iteration of the SDDM process requires using the scenario derived data model as a queryable resource of decision support information for consumption by an end-user. In the NDM-1 scenario, the following questions can now be answered by querying the data model:

-When did the NDM-1 events occur?

-In what locations around the globe did NDM-1 occur?

-What genetic variation in the NDM-1 gene was found?

Each of these questions can be answered by traversing the multi-relational directed graph starting at the scenario node. For example, to find the dates of all NDM-1 events, first follow the outgoing edges from Scenario:Scenario1003. If the node reached is of type Scenario: event, then report the date property. Otherwise, if it is of type outbreak, follow all outgoing edges to reach nodes of type Scenario:event and report their date (i.e. depth first search). Graph based traversals can be easily extended to support queries to find geo-location and genetic variation information on this NDM-1 data model. More complex queries are also possible using path algebra [27]. Sensors continuously deposit more RDF statements into the multi-relational directed graph as they analyze data streams. As the multi-relational directed graph gets updated, the traversal behaviour and results will change. This forms the basis for user interfaces.

### Traversing the generated model

From a technical perspective, traversing the model generated by SDDM requires constructing programs that crawl the linked data on the Semantic Web. Here we will illustrate how these programs can be constructed using the Gremlin programming language (https://github.com/tinkerpop/gremlin/wiki). Gremlin is a scripting language optimized for traversing graph structures, much the same way a person may navigate the web. Gremlin is built on top of the Groovy programming language which itself is built on top of Java. Java code and Groovy code will both work inside of Gremlin. Gremlin itself sits in a technology stack for interacting with graph databases that includes Pipes, Blueprints, Rexter, and many graph databases including Neo4J, OrientDB, DEX, and RDF Sail graphs. Gremlin is similar to SPARQL, it is a language for expressing queries over multi-relational graphs. Gremlin is functionally superior to SPARQL, but lacks the intuitive and simple syntax. Both Gremlin and SPARQL can easily be imbedded into software written in Java. Here we will limit the discussion of the Gremlin features to those queries alluded to in the previous section.

The first query considered in the previous section was when did the NDM-1 events occur? The following Gremlin code loads the scenario RDF model into memory, adds namespaces to increase usability and readability, seeds the start of the query at the Scenario1003 node (Node 1 in Figure 4) and then traverses the model. A list of common namespaces is in Table 1. The traversal starts at node 1 (represented as v in the script). The statement v.out(BioSITES:hasOutbreak) retrieves all of the nodes of type BioSITES:outbreak reachable from v. A statement of type v.out() gets the URI of the node, a statement of type v.outE() gets outgoing edges from v. These statements can be chained together to complete a query. An example of such a traversal is shown below.

**Table 1:** Namespaces and abbreviations used in the text.

Name	URI	Description	Reference
DBpedia	http://www.dbpedia.org/	Ontology of wikipedia entries	DBpedia: A Nucleus for a Web of Open Data
SO [sequence ontology]	http://www.sequenceontology.org/	Ontology of genetic sequences	The Sequence Ontology: a tool for the unification of genome annotations
GO [gene ontology]	http://www.geneontology.org/	Ontology of biological information	Gene ontology: tool for the unification of biology. The Gene Ontology Consortium
Bio2RDF	http://bio2rdf.org/	Converts biological info into RDF	Bio2RDF: Towards a mashup to build bioinformatics knowledge systems
Scenario	www.BioSITES.org/scenario	Ontology of the BioSITES terms	
Interpro	http://www.ebi.ac.uk/interpro/	Database of protein sequences	InterProScan – an integration platform for the signature-recognition methods in InterPro
ProMed	http://www.ProMedmail.org/	Collection of public health articles	ProMed-mail: An Early Warning System for Emerging Diseases
GeoNames	http://www.geonames.org/	Database of GPS locations around the world	Interlinking Open Data on the Web

Note, that the Semantic Web is easily traversed using the same techniques. For example to traverse the Bio2RDF repository from ’bio2rdf_interpro:IPR001036’ ,do:

This allows programs to update information when the local copy gets out of sync with the copy at Bio2RDF.

The second question, ‘in what locations around the globe did NDM-1 occur?’ is answered by a very similar traversal. The added .value asks Gremlin to display only the values on the property nodes and not the URI. This is also easily applied above to get the value for dates.

To answer the question, ‘what genetic variation in the NDM-1 gene was found?’ we wrote the following script that traverses the graph for protein sequences, and then does a system call to the Muscle multiple sequence aligner.

The output of this traversal is as follows:

We have only begun to scratch the surface of what is possible using Gremlin to traverse graphs. As a JVM language, Gremlin has the full power and flexibility of Java and Groovy to implement graph traversal algorithms. These algorithms can manipulate multiple graphs, traverse the Semantic Web, cache important subgraphs for use as catalogs, and process data streams.

## Discussion

The SDDM process was invented to solve a very daunting challenge; to be able to detect an outbreak as early as possible and perhaps even prevent it. Early detection requires the capacity to monitor data feeds such as online medical records, detectors such as JBAIDS, doctor’s blogs and social media for early indicators of an outbreak or a new disease. Prevention requires the ability to look deeper into the causative agents of the disease such as genetic components and quickly manufacture and deploy countermeasures. The capacity to do both these well is found in our ability to consume a massive amount of data as it is produced in real time, in our ability to integrate multiple sources of information of widely different types and our ability to understand the limitations and potentials of each data source.

The simple example presented here falls short of completely addressing this grand challenge in several respects. First, there is increasing evidence that the mainstream media (covered in the example via ProMED) can be quite slow to respond to an outbreak. For example, in the recent E. coli scare in Germany, ProMED sent it’s first alert on May 24, 2011, however the first cases occurred nearly a month earlier, Other sources of information are possibly better suited to early detection than ProMED and the mainstream media. While genetic sequencing has begun to be used as a diagnostic tool, it is not currently easily accessible globally. However, unlike PCR, sequencing will not miss subtleties such as signatures of genetic engineering or novel horizontally transferred genes. So, recognizing that they are not perfect, we chose these two feeds in our SDDM example because they represent very different data types, and therefore have different properties. From a technical perspective, a demonstrated capability to integrate data as divergent as free text and DNA sequence makes integration of more structured information such as medical records, output from diagnostic tools, and over the counter sales believable.

These data sources are not representative of all that is needed in bio-surveillance they are just two examples. The focus of this article is not to pit one data feed against another. Rather, the requirements of bio-surveillance will be met by simultaneous integration of tens or perhaps even hundreds of data feeds. SDDM is a technique that integrates multiple sources of information, each with different advantages, resolutions, timeliness, and capabilities. SDDM benefits from RDF as a robust and flexible data model for representing information of different types. RDF can effectively represent geographic positions, events, timelines, genetic features, people, social networks, publications, and even concepts extracted from free text. Another RDF advantage is that researchers in different fields can cooperate and build extremely complex models. SDDM takes advantage of these models using DBpedia, GeoNames, Bio2RDF, and other ontologies and resources on the Semantic Web. A second attribute of SDDM is that algorithms, processing methods and analytic routines are custom built. This allows the scenario writer to focus on the needs outlined in the scenario instead of building general purpose software that is overly complex and not based on meeting objectives. SDDM uses the idea of a scenario to reduce complexity. The scenario describes, in concrete terms, the goal of the integration. Elements outside of this goal are ignored. To be viewed as a success, a scenario driven integration need only meet the requirements specified in the scenario. It is up to the broader community to determine if the scenario meets their needs.

A common criticism regarding the use of scenarios is that they may help model past events, but ‘lightning never strikes the same place twice’. The argument is that scenarios help prepare us for something that will never happen again and will not prepare us for the next threat. The true power of this approach emerges when the collection of scenarios grows. It is built like Wikipedia, one article is not very useful, the true power of the platform is realized when thousands of articles are contributed. We can never anticipate every possible threat, but a library of known threats provides the capability to generalize. It may be possible to connect scenarios in unforeseen ways and thus build software that is broadly applicable. This is possible because causative agents will exist in the RDF graph (e.g. a toxin). A simple way to connect two scenarios is through a causative agent they both share (e.g. scenario A and B share the same toxin). Shortest path algorithms are one way to find if two elements are related in a graph. If two elements are related, then software can be generalized to detect both elements.

## Materials and methods

Application of SDDM to the NDM-1 example produces a scenario with detectors, catalogs, and sensors for the BioSITES system. Here elements of BioSITES are further described to provide context for how a scenario derived from SDDM would be processed.

### Software and techniques used in the streaming data kernel

Each component of the Streaming Data Kernel was coded in Java. Each component or program continually runs; when it receives a new message, it processes the message and then may send messages to other software components. ActiveMQ was used to connect each of the following BioSITES software components: Detectors, Sensors, Routers, and Controllers. Software component A is connected to software component B using publish-subscribe. Briefly, ActiveMQ implements publish-subscribe through the use of a broker. Brokers run continuosly. They can run on the same machine as the program that wishes to send messages, on the same machine where the program receiving messages resides or on a totally different machine. Programs that wish to send messages, send them to the broker using the ActiveMQ API. Programs that wish to receive messages connect to the broker and notify it that they wish to receive all messages sent by a particular program. ActiveMQ is a messaging protocol that allows components to be coded in many languages, including C++, .NET, Perl, Java, Python and Ruby.

Additional languages and technology were also used in the construction of the streaming data kernel, mainly due to programmer choice. The sequence read archive detector was written in Perl. It queries Entrez [28] on a regular schedule (currently every 24 hours – but this is variable) to find new submissions. When a new submission is found, it downloads the data and metadata (stored in XML) for each submission. The Perl program then calls a Java executable that uses JAXB (Table 2) to parse the contents of the XML-metadata into java objects, place the content of these objects into a message, contact the broker, and forward a message to downstream routers. This message also contains a URI corresponding to a local location where the raw sequence data is stored. Routers pick up this message and examine the metadata of the message and forward the message to sensors interested in reads of a specific type. The sequence read archive for example currently contains ‘Whole Genome Sequencing’, ‘Metagenomics’, ‘Transcriptome Analysis’, ‘Resequencing’, ‘Synthetic Genomes’, ‘Forensic or Paleo-genomics’, ‘Gene Regulation Study’, ‘Cancer Genomics’, ‘Population Genomics’, ‘RNASeq’, and ‘Other’. Sensors or analysis algorithms will only be interested in a subset of these study types, so they will subscribe only to those studies of interest through the ActiveMQ broker running on the routing machine. Sensors receive the message, download the raw sequence data, and begin computational analysis on it. In the case of the NDM-1 Blast sensor, the sensor has a copy of the NDM-1 sequences. When it receives a message that new sequences are available, it downloads a subset of the data, performs a system call to format the blast database on the new sequences, and then performs another system call to run Blast for all of the NDM-1 sequences against the newly formatted blast database. It then parses the output, and if there is a match, publishes the match to an outgoing ActiveMQ queue. Controllers that will render the advisory then consume this message. The NDM-1 sensor updates its copy of the NDM-1 sequences periodically by connecting to the server that contains the NDM-1 scenario through an ActiveMQ broker. When the server that contains the scenario receives the request, it performs a query to find all sequences on the server related to the NDM-1 catalog, and then sends the updated sequences back to the NDM-1 sensor in fasta format. A similar method is used when the sensor has found a new NDM-1 gene and would like to add it to the catalog on the scenario server.

**Table 2:** Technologies used in the SDDM process.

Technology name	Download site	Description
ActiveMQ	http://activemq.apache.org/	Used to connect each of the following BioSITES software components: Detectors, Sensors, Routers, and Controllers.
Rexter	https://github.com/tinkerpop/rexster/wiki/	Rexter exposes RDF over REST.
JAXB	http://jaxb.java.net/	Parses the contents of the XML-metadata into java objects, place the content of these objects into a message, contact the broker, and forward a message to downstream routers.
Neo4J	http://neo4j.org/download/	Used to import the ProMED mail archive for easy searching.
OrientDB	http://www.orientechnologies.com/orient-db.htm	A NoSQL DBMS which can store 150000 documents per second on common hardware
DEX	http://www.sparsity-technologies.com/dex.php	A high-performance *graph database* written in Java and C++ that allows the integration of multiple data sources
Jena’s TDB	http://www.openjena.org/TDB/	*TDB* is a component of *Jena*. It provides for large scale storage and query of RDF datasets using a pure Java engine
Jena	http://www.openjena.org/	An API for the manipulation of RDF data.
Sesame	http://www.openrdf.org/	An API for the manipulation of RDF
AllegroGraph	http://clos.org/agraph/downloads/	A system to load, store and query RDF data.
Virtuoso	http://virtuoso.openlinksw.com/dataspace/dav/wiki/Main/VOSDownload	Virtuoso is an innovative enterprise grade multi-model data server for agile enterprises & individuals.
Gremlin	https://github.com/tinkerpop/gremlin/wiki	Gremlin is a graph traversal language
Protege	http://protege.stanford.edu/	A free open-source Java tool providing an extensible architecture for the creation of customized knowledge-based applications
Tomcat	http://tomcat.apache.org/	Apache Tomcat is an open source software implementation of the Java Servlet and JavaServer Pages technologies.
SPARQL	http://www.w3.org/TR/rdf-sparql-query/	A relational like query interface to SQL Data

The ProMED data stream was constructed using the Groovy programming language. This program periodically looks at the sequential ID’s generated by the ProMED mail website when a new article is published. The ProMED mail detector forwards the article via an ActiveMQ broker where it is picked up by the ProMED NDM-1 sensor. This sensor has a copy of all ProMED mail articles relating to NDM-1. Currently, it does a pattern match on the incoming article to determine if it is similar to the NDM-1 articles in the reference catalog. If a match is found, it forwards an RDFized version of the article to the NDM-1 scenario server where it is added to the catalog. This consists of parsing the title, date, ProMED ID and content into RDF statements. It is not currently able to infer structure about entities in the article and create RDF statements from raw text.

### Hardware used

The BioSITES kernel is currently being run on a Linux system with 68 compute nodes, a management node, a backup management node and a data staging storage system. Each compute node has dual AMD Opteron 6134, 2.3 GHz processors (8 cores/CPU, 16 cores/node, 1088 cores/cluster). Each compute node has 64 GB DDR3 1333 MHz registered ECC memory (4 GB/core, 4.4 TB/cluster) and 4 x 2 TB disk drives (8TB/node, 544 TB/cluster). Data staging storage with 36 TB RAID array. Local storage on the nodes is currently 62% allocated, mostly by short read data and web data on the Hadoop filesystem. This system currently runs 2000 concurrent analysis algorithms or BioSITES ‘sensors’. These sensors have various functions, but a majority perform sequence analysis. The system also currently runs up to 64 concurrent ‘detectors’ that stream data for analysis to the BioSITES system. A single server in the same class as each node in the cluster is used to store our Bio2RDF mirror. Each running controllers are also running on the same hardware, 1 controller per instantiated scenario (e.g. the NDM-1 example outlined here has one running controller for serving RDF).

To contribute a scenario, or to add additional elements to the multi-relational graph as it is refined, a knowledgeable user need only use a personal computer and the Protégé software. Protégé can import external resources such as ontologies given they are of manageable size.

### Software and techniques used in the semantic catalogs

An RDF catalog is not all that different from HTML. RDF can be stored on files in the filesystem and then served by a webserver just like HTML content. For advanced queries, RDF can also be stored in a graph database. Examples include OrientDB, Neo4J, DEX, Jena’s TDB, AllegroGraph and Virtuoso (see Table 2). Graph database queries are executed in Sparql or Gremlin depending on the database. Bio2RDF consumes about 2 TB in size for a complete mirror, and each shard of the database is stored in a Virtuoso instance. Neo4J was used to import the ProMED mail archive for easy searching.

In the case of NDM-1, the scenario object was copied from the ontology editing computer and RDF triples where imported into a Neo4J sail store for access over Rexter. This allows a very small scenario model (i.e. about 80K) to interoperate seamlessly with Bio2RDF, GeoNames, and DBPedia infrastructures. Each of these resources provides SPARQL endpoints for querying and HTTP access for navigation. Graph traversals are implemented in Gremlin.

## Conclusion

The SDDM process uses a scenario to create software and a data representation. The result is a multi-relational directed graph containing the entities and relationships between entities required for the BioSITES system. This allows the BioSITES system to perform distributed, heterogeneous data integration in a manner not otherwise possible with traditional data modeling techniques.

This work provided an example where SDDM was successfully applied to complex data integration challenges. This example was chosen to be relatively simple for clarity. The process created a model of the emerging NDM-1 health threat, identified and filled gaps in that model, and constructed reliable software that monitored data streams based on the scenario derived multi-relational directed graph. Because of the complexity of the problem, the SDDM process significantly reduced the software requirements phase by letting the scenario and resulting multi-relational directed graph define what is possible and hence set the scope of the user requirements. Approaches like SDDM will be critical to the future of data intensive, data-driven science because they automate the process of converting massive data streams into semantic graphs or computable knowledge.

## Competing interests

The authors declare that they have no competing interests.

## Authors' contributions

DQ and TB conceived of the idea of SDDM. DQ, TB and SG constructed the Scenario. DQ designed and built the Scenario ontology. DQ and SG built the RDF graph for NDM-1. DQ wrote the content for the article. All authors reviewed, edited, and contributed to the final manuscript.

## Glossary

Annotation- is a note that is made while reading any form of text.

BioSITES- is a comprehensive program to develop a secure, authoritative, predictive, and complete reference standard for biological threat mitigation that will support detection R&D and lead to near real-time bio-surveillance.

Catalog- A catalog is a collection of objects from a data repository. BioSITES catalogs are constructed by making a Scenario:CatalogX node and then connecting that node to other nodes on the Semantic Web.

Data Mashup or Mashup - a purpose driven, customized data integration that facilitates question answering on a topic of interest.

Edge – the connection between two nodes in a graph is called an edge.

Event- single incidents that happen at a determinable time and location.

Namespace- A namespace name is a uniform resource identifier (URI). Typically, the URI chosen for the namespace of a given XML vocabulary describes a resource under the control of the author or organization defining the vocabulary, such as a URL for the author's Web server. In RDF and OWL namespaces are abbreviated namespace:content, where namespace is a URI prefix, and content is the ontology term or individual data item.

NDM-1- is an enzyme that makes bacteria resistant to a broad range of beta-lactam antibiotics. These include the antibiotics of the carbapenem family, which are a mainstay for the treatment of antibiotic-resistant bacterial infections. The gene for NDM-1 is one member of a large gene family that encodes beta-lactamase enzymes called carbapenemases. Bacteria that produce carbapenemases are often referred to in the news media as "superbugs" because infections caused by them are difficult to treat. Such bacteria are usually susceptible only to polymyxins and tigecycline.

Node – Graphs are constructed from nodes and edges. Nodes represent data or ontology classes. Edges connect nodes together in the construction of a graph.

Object – Also called an RDF individual, this is a specific instance of a data element. In multi-relational directed graphs, nodes represent objects.

Ontology- In computer science and information science, an ontology is a formal representation of knowledge as a set of concepts within a domain, and the relationships between those concepts. It is used to reason about the entities within that domain, and may be used to describe the domain.

Outbreak- are occurrences of a disease/infection greater than would be expected in an area.

OWL- The Web Ontology Language (OWL) is a family of knowledge representation languages for authoring ontologies. The languages are characterised by formal semantics and RDF/XML-based serializations for the Semantic Web. OWL is endorsed by the World Wide Web Consortium (W3C).

Property – An ‘edge’ in a multi-relational directed graph. This edge has a relationship type (i.e. a type of property).

Protégé’- is a free, open source ontology editor for editing RDF, OWL and other resources on the Semantic Web.

RDF- The Resource Description Framework (RDF) is a family of World Wide Web Consortium (W3C) specifications originally designed as a metadata data model. It has come to be used as a general method for conceptual description or modelling of information that is implemented in web resources, using a variety of syntax formats.

RDFS – The Resource Description Framework Schema.

RDFizers – Software that reads traditional data representations such as relational databases, text files, and data streams and converts the content into a multi-relational directed graph. RDFizers do not always perform rigorous analysis, so information can be lost in the process.

Scenario- For BioSITES, a scenario is a structured description of a malicious action or series of actions that causes harm in health, economics, or quality of life.

SDDM- Scenario Driven Data Modeling.

Semantic Web- is a "web of data" that enables machines to understand the semantics, or meaning, of information on the World Wide Web.
